# Optimizing the performance of a reactor by reducing the retention time and addition of glycerin for anaerobically digesting manure

**DOI:** 10.1080/09593330.2014.983989

**Published:** 2014-12-13

**Authors:** Maikel Timmerman, Els Schuman, Miriam van Eekert, Johan van Riel

**Affiliations:** ^a^ Wageningen UR Livestock Research, P.O. Box 338, 6700AHWageningen, The Netherlands; ^b^ LeAF, P.O. Box 500, 6700AMWageningen, The Netherlands

**Keywords:** manure, biogas, retention time, glycerin, optimization

## Abstract

Anaerobic digestion of manure is a widely accepted technology for energy production. However, only a minimal portion of the manure production in the EU is anaerobically digested and occurs predominantly in codigestion plants. There is substantial potential for biogas plants that primarily operate on manure (>90%); however, the methane yields of manure are less compared to coproducts, which is one of the reasons for manure-based biogas plants often being economically non-viable. Therefore, it is essential to begin increasing the efficiency of these biogas plants. This study investigated the effect of decreasing retention time and introducing a moderate amount of glycerin on the biogas production as methods to improve efficiency. An experiment has been conducted with two different manure types in four biogas reactors. The results of the study demonstrated that, first, it was possible to decrease the retention time to 10–15 days; however, the effect on biogas production varied per manure type. Secondly, the biogas production almost triples at a retention time of 15.6 days with an addition of 4% glycerin. The relative production-enhancing effect of glycerin did not vary significantly with both manure types. However, the absolute production-enhancing effect of glycerin differed per manure type since the biogas production per gram VS differed per manure type. Thirdly, the positive effect of the glycerin input declines with shorter retention times. Therefore, the effect of glycerin addition depends on the manure type and retention time.

## 1. Introduction

In 2011, the annual production of manure (cattle, swine and poultry) was estimated to be approximately 1400 million tonnes within the combined member states of the European Union (EU). Cattle are the largest producer of manure with an annual production of almost 1100 million tonnes wherein around 500 million tonnes is in the form of liquid manure (slurry and source separated liquid). Swine are the second largest producer with an annual production of about 175 million tonnes which comprises around 150 million tonnes of liquid manure (slurry and source separated liquid). In total, 50 million tonnes of livestock manure were anaerobically digested in 5250 biogas plants (4700 farm size biogas plants) which primarily operate with mesophile temperature ranges.[1] Thus, only a minimal amount of annual manure production in the EU is being exploited for energy production in biogas plants. Therefore, significant potential for increase in energy production in the EU from manure is plausible. For liquid manure, anaerobic digestion is most likely the most appropriate technology for producing renewable energy in the form of biogas that can be directly employed for the production of electricity and heat in a combined heat power (CHP) plant or ameliorated as a substitute for natural gas.

Most full-scale biogas plants operate with manure with as well as with a substantial amount of coproducts in the form of (energy) crops, crop residues and by-products from the feed and food industry in order to make the energy production economically feasible. Due to the magnitude of these codigestion plants, operation generally requires half to full-time employment. A significant number of farmers are interested in utilizing the energy that is made available from the manure that is produced on their farm, but do not want to use substantial amounts of coproducts. However, to ensure the economic viability of such an operation, the work load related to ‘maintenance and operation’ should be minimized; therefore, the biogas plant should operate primarily from the manure produced on site. However, under the current circumstances, the financial returns of these manure-based biogas plants are generally very insufficient. The investment costs are high and, the biogas production from manure is also less compared with the biogas production from energy crops and coproducts from the feed and food industry. For example, a general guideline for the biogas yield of cattle manure is 17.8 Nm^3^/ton (8.9% VS with 0.20 Nm^3^/kg VS) and 19.5 Nm^3^ (6.5%VS with 0.30 Nm^3^/kg VS) for swine manure, which are substantially less than the biogas yield of coproducts such as molasses (190 Nm^3^/ton) or non-food grains (156 Nm^3^/ton).[2] Therefore, knowledge development is necessary in order to improve the cost-effectiveness and profitability of small-scale manure-based biogas plants which, in return, will afford an opportunity to increase the renewable energy production in the EU that is created from manure.

The focus in this study was to increase the knowledge about the following measures in order to improve cost-effectiveness from manure-based biogas plants:

- Decreasing the retention time will lead to a more significant throughput (greater amounts per day) of manure digested in the same size reactor. Alternatively, the reactor can be constructed more moderately for the same throughput. This will reduce investment costs. A prerequisite is that biogas production from manure should remain at stable, acceptable levels.
- Introducing a moderate amount of a liquid coproduct will perform as an enhancer to increase biogas production. A liquid coproduct is easily stored in a tank from which the coproduct can be pumped through a pipeline to the biogas plant. This significantly reduces the amount of labour required for operating the biogas plant while still providing the benefit of substantially increased biogas production from the same sized biogas plant which will subsequently result in greater financial returns.

The objective of this study was to research the single and combined effect of reducing the retention time of manure and introducing a minimal amount of coproduct into the biogas production. An adaptive dynamic linear model was employed for the online estimation of biogas response to manure and glycerin input. The parameters in this model may change over time and is updated in accordance with a Bayesian approach for online analysis of time series.[3] The primary utilization of this dynamic model consists in its ability to determine economically optimal settings for daily input levels of a manure-based biogas plant.

## 2. Materials and methods

### 2.1. Manure, digestate and glycerin

Two manure types were exploited. The first type was swine manure that was obtained at the Swine Innovation Centre in Sterksel, the Netherlands. The delivered swine manure did not originate at the Swine Innovation Centre but from other swine farms. The biogas production was less (200–250 ml/g VS) than expected when based on literature values.[2,4] Unfortunately, the manure was delivered by an intermediate company, and the data of origin of the swine manure could not be retrieved. This made it impossible to establish what type of diet was provided to the swine which could, at least partially, have explained the observed low biogas production from the manure. The manure could also have been garnered for a longer period of time. The second type of manure originated from the Knowledge Transfer Centre De Marke in Hengelo, the Netherlands, and was dairy cattle manure producing 200–300 ml biogas/g VS, which is in accordance with values reported for cattle manure.[2,3] The dairy cattle were provided with a roughage diet of 50% grass and 50% maize on a dry matter (DM) basis with supplement concentrates of 7 kg per cow per day. Several times during the experiment, both manures were collected, transported to the laboratory, and maintained in a cold store. Glycerin was employed as the liquid coproduct in the experiment due to its anticipated elevated biogas production per ton. Due to the moderate amount of glycerin required, this was collected only once at one of the storage tanks from the full-scale biogas plant at the Swine Innovation Centre in Sterksel, the Netherlands. Digestate (8.6 kg) from the Microferm biogas plant at the Swine Innovation Centre in Sterksel was used to activate the reactors containing the swine manure. Digestate (8.8 kg) from the biogas plant at the Knowledge Transfer Centre De Marke in Hengelo, the Netherlands, was employed to activate the reactors containing dairy cattle manure. The composition of both manure types, the digestates and the glycerin is illustrated in Table 1 (for more details refer to Appendix 1).

**Table 1.** Composition of the manures, glycerin and inoculum digestates (ID) used to start up the biogas reactors.

	Swine manure		Dairy cattle manure		Glycerin
Parameter	ID	Feed	ID	Feed	
pH	8.1	7.5 ± 0.3	7.8	7.5 ± 0.3	8.1
TS (g/kg)	54.0	77.8 ± 6.0	53.2	83.5 ± 4.9	885.9
VS (g/kg)	37.6	56.8 ± 3.5	40.0	65.9 ± 4.0	842.1
VS/TS (%)	69.6	73.2 ± 3.2	75.2	78.9 ± 0.5	95.0
COD_t_ (g/kg)	52.1	81.5 ± 5.0	61.7	101.6 ± 2.7	1185
VFA (g VFA-COD/kg)	6.7	44.5 ± 27.4	16.0	123.5 ± 29.8	40.5
N_t_ (g/kg)	5.3	6.2 ± 1.2	3.5	3.7 ± 0.1	0.8
NH_4_-N (g/kg)	3.9	4.1 ± 0.8	2.0	1.7 ± 0.0	<0.1
Density (g/l)	1024	1036 ± 12	1017	994 ± 18	1351

Notes: All analysis have been carried out in duplicate. Feed was delivered in five different loads for each manure type. Values are means and standard deviations.

Variations in manure composition were ascertained during the experiment; however, the standard deviation of the samples generally remained within 10% of the overall average. The VFA content of the manure was the least constant parameter for both manure types. This was primarily caused by the second batch of each manure that differed from the other batches.

### 2.2. Operation of the biogas reactors

The experiments were conducted in a laboratory with four biogas reactors creating a net working volume of 9 l per reactor. The reactors were continuously mixed (60 rpm) and maintained at a constant temperature of 37°C with a water-jacket. The reactors were fed twice a week (Monday morning and Thursday afternoon). Due to the practical limitations and restrictions of the laboratory, it was not possible to feed three times or more per week. Prior to each feed, the required amount of digestate (pre-calculated to achieve the desired retention time, see below) was extracted from the reactors by pumping and subsequently replacing with an equal amount of fresh manure. The biogas production was continuously measured using Milligascounters (Ritter, Bochum, Germany). If necessary, two counters were juxtaposed and connected to ensure sufficient recording capacity for each individual reactor. It was substantiated that the biogas production did not exceed the capacity of the Milligascounters (data not shown). The biogas production data were logged continuously. The biogas composition was determined twice a week prior to remove the digestate before every new feed. Also, at that point in time, digestate samples were extracted for analysis of pH, VFA, and NH_4_-N.

During the first 28 days, the reactors were started up, after which one reactor was established as a monodigestion (R1 and R3) and a second reactor as a codigestion (R2 and R4) for each manure type. After 74 days, reactor R1 (swine manure) and reactor R3 (dairy cattle manure) were switched to codigestion until completion of the experiment on day 143. After 112 days, reactor R2 (swine manure) and reactor R4 (dairy cattle manure) were switched to monodigestion until the end of the experiment on day 143.

An adaptive dynamic model was utilized in the experiment in order to establish a response curve to input for each feeding point in time. This time series analysis was in accordance with a Bayesian approach and was proposed by West and Harrison.[5] A dynamic model consists of an observation equation and a system equation. The observation equation is a linear regression model describing the relationship between biogas production and the input of manure and glycerin. The parameters in this equation are time dependent. The substrate of a manure-based biogas plant can change over time; therefore, the response to input will also change. The online estimated response curve was utilized as an operational tool for adjusting the feeding strategy (increasing or decreasing the input) separately for each reactor during the execution of the experiment. In the first phase of the experiment, an increase was only advised if the response curve exhibited an increased biogas production with a greater input. After each feeding point over time, the database was updated with the new measurements and the response curve was again re-evaluated with the adaptive dynamic model. Therefore, the dynamic model was primarily employed in this experiment as a feedback system to ascertain when the microbial biomass was able to convert a greater input (only manure or combined with glycerin) into more biogas.

During the first week of operation, the input was increased to a total input of 1500 g per reactor per feed which resulted in a retention time of 20 days. This input remained consistent during the following 2.5 weeks after which the input was again increased based on the response curve. At the completion of the experiment (particularly the last two weeks), the input was no longer increased based on the response curve; however, the input was increased to determine whether the biogas production would decrease when retention time was decreased.

### 2.3. Statistical analysis

By using time series modelling in a mixed model (REML), the data of the reactors were analysed in one longitudinal model whereby the average reactions (over the reactors and experimental period) of the adjustments in manure input (retention time) and glycerin input were estimated. For the response of the glycerin input, the effect was modelled in such a way that the change in glycerin input was also an influence (due to growth of bacterial population). The interaction between glycerin input and manure input was also estimated. The model (on a log scale) after backwards transformation describing the biogas production is illustrated by

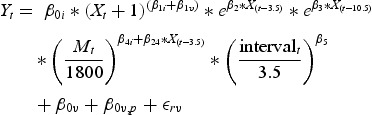

With:

1. Measured biogas production (in ml) during the time interval of two feeds over time.
2. Estimated biogas production at the standard manure input (1800 g) per manure type *i* =1 (dairy cattle manure) or i=2 (swine manure)
3. Glycerin input (g) preceding each time interval of biogas measurement
4. Response parameter of the effect of the most recent glycerin input (per manure type *i*)
5. Random effect differences between reactors for the reaction on glycerine *X*_*(t*−3.5*)*_ and *X*_*(t*−10.5*)*_ Glycerin inputs of the preceding points in time at respectively 3.5 and 10.5 days preceding to the current feeding
6. Response parameter of the effect of the preceding glycerin inputs at respectively 3.5 and 10.5 days preceding the current feeding
7. Manure input (g) preceding to the time interval of biogas measurement
8. Response parameter of the effect of manure input (per manure type *i*) in which the effect is expressed per unit of relative change (manure input/1800)
9. Interaction-effect of manure input preceding the time interval of biogas measurement and glycerin input in the second last feeding (3.5 days before)
10. Time (day) between moment of measurement of the biogas and the preceding biogas measurement
11. Correction factor for differences in the time interval between feedings and moment of biogas production measurement
12. Random level differences in biogas production between reactors
13. Random level differences in biogas production between batches of manure (within reactors)
14. Random day effect within reactors (=residual variance)

The mixed model analysis was performed in Genstat with the procedure REML. The analysis accounted for the dependence between repeated observations in time per reactor by estimating the factor for autocorrelation. In the analysis, the first two weeks were not considered due the start up of the reactors. Furthermore, the presupposition was made that, for each manure type, both reactors reacted identically since the analysis from the mixed model demonstrated that a reactor effect was not evidenced for the entire test period.

### 2.4. Analysis of manure, digestate and biogas

Total solids (TS) and volatile solids (VS) content of the manure as well as digestate were conducted according to the Standard Methods.[6] The pH was measured with a daily calibrated electrode. Chemical oxygen demand (COD), total nitrogen (N_t_), and ammonium-nitrogen (NH_4_-N) content were also determined according to the Standard Methods,[6] but selenium instead of copper was exploited as a catalyst in the N_t_ analysis. For the volatile fatty acids (VFAs) content analysis, the samples were centrifuged for 10 min at 10000 rpm. The supernatant was then diluted in 3% formic acid and centrifuged again (10 min at 10000 rpm) (final concentration in the sample was 1.5%). The VFA analysis was performed by gas chromatography (GC) (HP 5890 GC) with the GC-packed technique. The column is glass (2 m*6 mm*2 mm) that is packed with 10% Fluorad 431 on Supelco-port 100–120 mesh. The oven temperature was 130°C, and the carrier gas was N_2_ saturated with formic acid at a flow of 40 ml/min. The injector temperature was established at 200°C and the flame ionization detector at 280°C. The sample size comprised 1 µl. The detection limit for VFA analysis was 20 mg/l for each separate VFA (C_2_–C_5_).

The biogas composition was measured with the GC-wide bore technique (Shimadzu; GC 2010) including loop injection. The columns that were utilized were Porabond Q (50 m×0.53 mm; 10 µm, Varian; Part. no.CP7355) and Molsieve 5A (25 m×0.53 mm; 50 µm, Varian; Part.no. CP7538), and the columns were juxtaposed and connected. The oven temperature was 75°C, the carrier gas He and the column pressure 1.0 bar. The inlet temperature was 120°C; the thermal conductivity detector was at 150°C. The sample size incorporated 50 µl. The calibration gas consisted of CH_4_: 50.11%, CO_2_: 24.8%, N_2_: 20.6%, O_2_: 2.97% and H_2_: 1.52%.

## 3. Results

### 3.1. Start up of the biogas reactors

**Figure 1.**
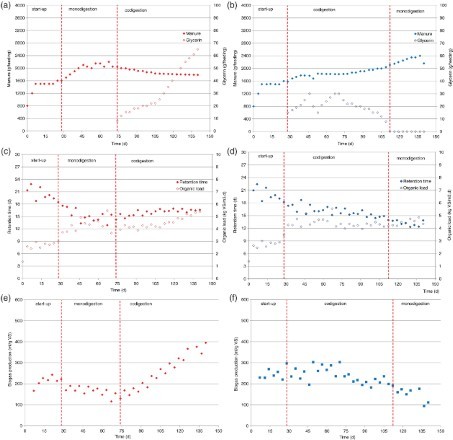
Performance of reactors fed with swine manure. (a) Input R1; (b) input R2; (c) retention time and organic load R1; (d) retention time and organic load R2; (e) biogas production R1 and (f) biogas production R2.

**Figure 2.**
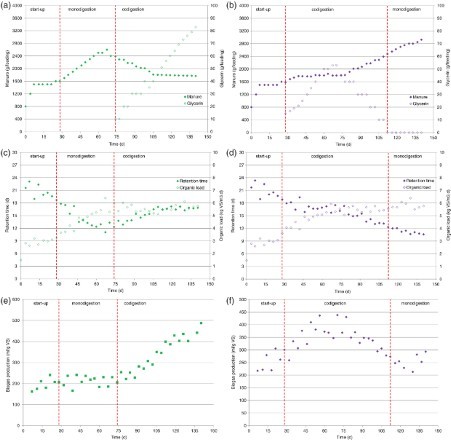
Performance of reactors fed with dairy cattle manure. (a) Input R3; (b) input R4; (c) retention time and organic load R3; (d) retention time and organic load R4; (e) biogas production R3 and (f) biogas production R4.

During the start-up period (the first 28 days), the four biogas reactors were operated in a similar manner aiming for a retention time of 20 days for each reactor (Figures 1 and 2). The duplicate reactors that were supplied with swine manure exhibited similar performance results. Those fed with manure from dairy cattle demonstrated insignificant variances resulting in more substantial biogas production in reactor R4 (250–300 ml/g VS) than in reactor R3 (approximately 200 ml biogas/g VS) on day 28 following the start-up. The experiment was continued as the biogas production of reactor R3 remained at a level that was expected prior to the experiment. A direct cause for the difference between both reactors was not determined. The VFA content (<1000 mg VFA-COD/l) and ammonium concentration (1500 mg NH_4_-N/l) were similar in both reactors. The COD:N-ratio of the input was about 17 for the swine manure reactors and 27 for the dairy cattle manure reactors during this period (Appendices 2 and 3). The trends for the most important parameters of the reactors are depicted in Table 2.

**Table 2.** Summary of the most important parameters of the biogas reactors.

		R1		R2	
Swine manure	Start-up	Mono	Co	Co	mono
RT (days)	∼20	20→15	15→20	20→15	15→10
COD/N-ratio	17–18	12–13	→20	∼15	→12–13
Biogas production (ml/g VS)	200–250	200→150	→400	∼200	→100
VFA (VFA-COD/l)	<500	→1500	→4500	→4000→1000	→<500
C_2_/C_3_	C2	C2	C2/C3 = 1	Variable	C2
NH_4_-N (mg/l)	1500–2000	2000→3000	2500/3000	2000/2500	2000/2500
		R3		R4	
Dairy cattle manure	Start-up	Mono	Co	Co	mono
RT (days)	∼20	20→10	10→20	20→12	→10
COD/N-ratio	25–30	28–30	→45	→40→27	→25–30
Biogas production (ml/g VS)	200–300	200	→500	→450→250	→250
VFA (VFA-COD/l)	<1000	→1500	→5000→1000	→5000→500	→<500
C_2_/C_3_	C2	→C2/C3 = 2	Variable, C3 > C2	C2/C3∼1	C2/C3∼1
NH_4_-N (mg/l)	1500–2000	∼1000–1500	∼1000–1500	∼1000–1500	∼1000–1500

Note: RT = retention time, C2/C3 = ratio acetic acid (C2) and propionic acid (C3).

### 3.2. Swine manure reactors

Following the start-up period, monodigestion was applied in reactor R1. The retention time during this period of monodigestion was gradually reduced to 15 days (Figure 1). It was not possible to decrease the retention time any further. The biogas production per gram of VS initially slightly increased but, towards the completion of the monodigestion period, it again decreased to its initial level. This concurred with an increase in VFA and ammonium levels in the digestate (Appendix 2); however, levels remained within acceptable limits. Most likely, the quality of the manure as a whole is an explanation for the decreased biogas production. From day 75, glycerin was introduced to reactor R1, and the reactor operated as a codigestion process. The glycerin dose was gradually increased to a maximum of 3.6% (w/w) which resulted in a concurring increase in the biogas production. The entire biogas production had a linear correlation to the glycerin dose and achieved a level of 395 ml/g VS added and a volumetric methane yield of 1.50 m^3^ CH_4_/m^3^_reactor_ per day. Towards the completion of the test period, the VFA concentration in the reactor increased to approximately 4500 mg VFA-COD/l, and the production pattern shifted from acetate towards propionate which effectually suggests that the biogas-forming methanogens were no longer capable of maintaining a pace with the loading rate. The pH remained stable throughout the test period (pH 8.1 ± 0.1); therefore, acidification of the reactor did not occur. The methane content of the biogas was 68 ± 3%.

Reactor R2 was supplied with a gradually increasing glycerin concentration (maximum 1.7% w/w) up to day 50. The total biogas production attained a level of 303 ml/g VS added and a volumetric methane yield of 0.85 m^3^ CH_4_/m^3^_reactor_ per day. Thereafter, the glycerin addition was decreased until day 112. Dissimilar to the events in reactor R1, the addition of glycerin did not result in an increase of biogas production (Figure 1, Table 2). There was an insignificant increase in the VFA to 400 mg VFA-COD/l; however, the gradual decrease in glycerin addition resulted in the consumption of VFA and a shift from propionate to acetate (Appendix 2). The transition to monodigestion of the manure after day 112 did not result in a more extensive transformation of VS in the manure to methane (Table 3). For both reactors R1 and R2, the amount of VS transformed during digestion was stable or decreased over time. The pH remained almost constant at approximately 8.1 ± 0.1 regardless of the method of operation. The methane content of the biogas in reactor R3 was 68 ± 2%.

**Table 3.** Percentage volatile solids (%VS) of the manure that is converted during different modes of operation (S = start-up period; M = monodigestion, C = codigestion ) in the experimental period.

Swine manure						Dairy cattle manure					
R1			R2			R3			R4		
Time (days)		% VS	Time (days)		% VS	Time (days)		% VS	Time (days)		% VS
0–28	S	22	0–28	S	25	0–28	S	20	0–28	S	26
28–74	M	20	28–112	C	21	28–74	M	26	28–112	C	35
74–144	C	19	74–144	M	18	74–144	C	30	74–144	M	30

Notes: Full conversion of the glycerin, with 10% of glycerin-COD being used for bacterial growth and 90% conversion to methane is assumed. The remaining amount of methane is then converted back to VS. Assumed was a conversion-factor of 1.4 g COD/g VS.

### 3.3. Dairy cattle manure reactors

Following the start-up period, monodigestion was applied in reactor R3. The retention time during this period of monodigestion was gradually reduced to 10–12 days. The total biogas production increased during this period while the biogas production per gram VS remained somewhat constant. Due to the decrease in retention time, the VFAs concentration increased slightly just prior to the transition from monodigestion to codigestion in this reactor on day 75. From day 75 onwards, codigestion was applied, and the glycerin dose was slowly increased to a maximum of 4.7%-(w/w) on the last day of the experimental period. The entire biogas attained a level of 487 ml/g VS added and a volumetric methane yield of 1.91 m^3^ CH_4_/m^3^_reactor_ per day. Directly following the first input of glycerine, the VFA concentration increased to 5000 mg VFA-COD/l; however, this was most likely related to the substantial organic load applied at the end of the period of monodigestion preceding this codigestion stage. Around day 105, the VFA concentration had decreased to below 1000 mg VFA-COD/l which was possibly a result of the increase in the retention time to 16 days during this period. The biogas production increased during this period to more than 400 ml/g OM. The pH remained almost constant at approximately 7.9 ± 0.1 regardless of the method of operation. The methane content of the biogas in reactor R3 was 68 ± 3%.

Following the start-up period, codigestion was applied in reactor R4, and glycerin was added whereby the amount of glycerin in the feed was gradually increased. The maximum amount of glycerin applied was 2.7%-(w/w) on day 70. Between day 28 and day 70, a gradual increase in biogas production consequently occurred due to the greater input of glycerin. Around day 70, the biogas production attained a level of 438 ml/g VS added was established with a volumetric methane yield of 1.68 m^3^ CH_4_/m^3^_reactor_ per day. During this period, the VFA concentration also increased (with a one time maximum of approximately 5000 mg VFA-COD/l). However, the microbial population in the reactor was simultaneously slowly shifting considering the fact that, before day 60, the C3 concentration was higher than the C2 concentration. Shortly after this period, the VFA concentration decreased to below 1000 mg VFA-COD/l. After day 70 to day 112, the amount of glycerin in the feed was gradually reduced while the input of manure was increased. From day 112, monodigestion was applied, and the retention time was further reduced to around 10 days. The biogas production, and the VFA concentration remained stable. However, the biogas production expressed per gram VS was greater at a retention time of 10 days than was the level of biogas production during start-up (with a retention time of 20 days). The pH in reactor R4 remained almost constant at approximately 7.9 ± 0.1 regardless of the method of operation. The methane content of the biogas in reactor R4 was 68 ± 4%.

A drastic decrease in the retention time of the dairy cattle manure reactors was feasible. In general, this led to an increase in the VFA concentration in the digestate, but the reactors appeared to be able to manage this increased level. This may also have been related to the fact that the ammonium concentration in the digestate remained low (below 1500 mg NH_4_-N/l, Table 2) throughout the experimental period. Results (Table 3) suggest that the input of glycerin has had a positive effect on the conversion of the VS in the dairy cattle manure; however, this must be investigated in more detail since both reactors experienced a different start-up level. Nevertheless, it is clear that the input of glycerin did not result in a decreased VS conversion.

### 3.4. Statistical analysis

The parameters for the mixed model have been estimated for both manure types (Table 4). The results of the parameter estimations demonstrate that the reduction of the retention time (by increase in manure dose) leads to an increased biogas production for dairy cattle manure. Furthermore, it indicated that the relative production increase for both manure types is similar and not significantly dissimilar (Table 5).

**Table 4.** Parameter estimations for direct response on glycerin and manure for testing of differences between manure type.

	Model parameter				
Parameter description		Swine manure	Dairy cattle manure	SED	Sign.
Biogas production at standard dose (monodigestion)	*β*_oi_	9.84^b^	10.23^a^	0.12	***
Effect of glycerin dose-increase	*β*_1i_	0.082	0.069	0.024	NS
Effect of manure dose-increase	*β*_4i_	0.27^b^	1.11^a^	0.35	*

Note: Parameter estimates with different superscripts are significantly different. *p < 0.05, **p < 0.01, ***p < 0.001.

**Table 5.** Parameter estimations for delayed response on glycerin dose and interaction with manure dose.

	Model parameter			
Parameter description		Value	SE	Sign.
Effect of glycerin dose in second last feeding	*β*_2_	0.006	0.002	***
Interaction effect of glycerin dose in second last feeding and manure dose in last feeding	*β*_24_	−0.027	0.011	*
Effect of glycerin dose in feeding of 10.5 days before last feeding	*β*_3_	0.004	0.002	*
Correction factor because of variation in time interval between moment of feeding and moment of measurement of biogas production	*β*_5_	0.839	0.0049	***

Note: *p < 0.05, **p < 0.01, ***p < 0.001.

**Figure 3.**
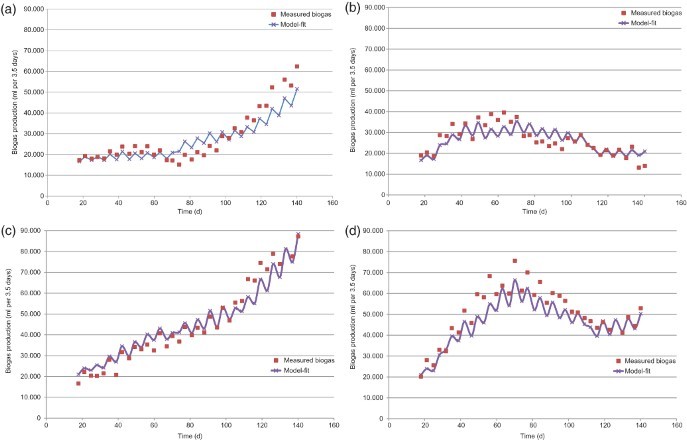
Measured biogas production (in ml per 3.5 days) during the experimental period (in days), including the model fit on basis of the estimated model parameters in the mixed model. (a) R1; (b) R2; (c) R3 and (d) R4.

The results of the parameter estimations depict that the glycerin level of the second last feeding positively affects the biogas production of the current feeding. This suggests that the glycerin of the second last feeding is not yet fully converted at 3.5 days after addition to the reactor. The interaction effect of the glycerin dose in the second last feeding with the manure dose in the last feeding is also significant. An explanation could possibly be the elimination of unconverted VS in the digestate when the retention time is decreased. Furthermore, a positive effect was ascertained for the glycerin dose of 10.5 days (3 feedings) before the last feeding. This could be the result of a positive long-term effect of glycerin on the growth of the bacterial biomass. Naturally, the correction factor for the differences in the time interval between the time of feeding and the time of the biogas measurement is strongly significant. Figure 3 illustrates the measurements of the produced biogas per reactor. It also depicts the model fit of the mixed model (line). Results of the parameter estimations indicate that, in this experiment, the standard amount of input results in more biogas being produced from dairy cattle manure when compared with swine manure.

**Figure 4.**
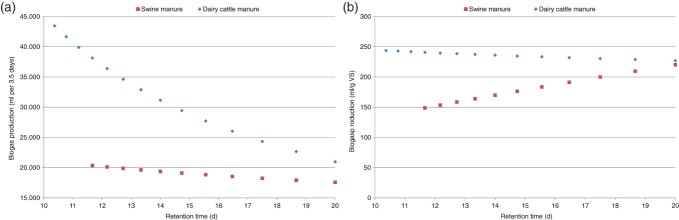
Estimated biogas production at specific retention times. (a) In ml per 3.5 days and (b) in ml per gram VS.

The specific design of the mixed model affords an opportunity to demonstrate the effects of retention time and glycerin level in the input. By employing the method of feeding based on the dose–response method, it is plausible to decrease the retention time of dairy cattle manure to approximately 10 days (Figure 4). While doing this, the biogas production per gram VS remained constant.

**Figure 5.**
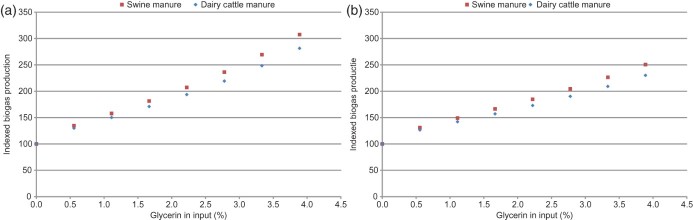
Estimated increase in biogas production (index rating) at specific glycerin percentages in the input. (a) Retention time of 15.6 days and (b) retention time of 14 days.

A moderate dose of glycerin resulted in a threefold increase in the biogas production (Figure 5) with approximately 4% (w/w) of glycerin in the feed at a retention time of 15.6 days. The differences in the production-enhancing effect of the glycerin for both manure types did not vary significantly. According to the model estimations, the effect of glycerin decreases in correlation with the lower retention times as the efficiency of glycerin is dependent upon manure quality as well as retention time. This can be concluded from the fact that glycerin addition resulted in absolute greater biogas yields with dairy cattle manure for the complete range of the tested retention times.

## 4. Discussion

The entire content of VFAs and the ratio of the VFAs fluctuated per reactor. At various points in time, the input was the same, however, the content of VFAs significantly differed between these points. In reactor R4, temporary reduction of the input of glycerin resulted in an instantaneous decrease in the VFA content. However, a subsequent increase in the glycerin input did not result in a renewed increase in the VFA content. The buffer capacity of the reactors remained somewhat constant during the experiment (Appendices 2 and 3) as did the pH. The content of VFAs alone is, therefore, not a measure of the effects of retention time or organic load that is possible in a reactor. It demonstrates that certain groups of microorganisms are more active than others which subsequently indicates the activity of different groups of microorganisms during digestion and whether or not there is a balance between the different microbial groups involved in the methanization of complex VS at a certain point in time. In the event of an imbalance, elevated concentrations of VFAs in the digestate is a probability. In practice, care should be taken to avoid undesirable methane emission in the post-digestion storage, which is a result of the conversion of high VFAs, by also capturing the biogas produced in the post-digestion storage.

The results of the monodigestion indicate that it is possible to anaerobically digest swine manure in a retention time of 12–14 days at an organic load of 4.5–5.0 kg VS/m^3^ and dairy cattle manure in a retention time of 10–12 days at an organic load of 6.0–6.5 kg VS/m^3^. However, the effect of the brief retention times on the biogas production differed per manure type. The biogas production per gram VS of swine manure decreased while the biogas production of dairy cattle manure remained constant which resulted in an absolute increase in the total biogas produced as a result of the greater throughput of the dairy cattle manure. The ammonium content of the dairy cattle manure was 2.5 g/l, which is considerably less than the 5.0 g/l of the swine manure. Previous research [7,8] with anaerobic digestion of fresh swine manure and an ammonium content less than 4.0 g/l demonstrated a greater biogas production with a retention time of 15 days. The elevated ammonia content possibly incited ammonia inhibition of the process which resulted in diminished biogas production.[9,10] According to Braun,[4] a minimum retention time of 20 days is required for anaerobic digestion of swine and cattle manure. Other literature indicates that retention times of 10–15 days are feasible for swine [11–13] and cattle manure,[14–16] however, the conditions under which the research has been performed vary extensively (e.g. diluted manure, volatile solids content, organic load, reactor configuration, temperature, etc.). Therefore, it is difficult to compare research results, but anaerobic digestion of liquid manure at retention times of 10–15 days is generally possible with the biogas production close to the maximum BMP reported in literature under the condition that the manure is of good quality (e.g. fresh, low ammonia content, etc.) and good operational management of the reactor occurs.

The addition of a moderate amount of glycerin led to a substantial increase in biogas production, however, the effect varied per reactor. The response of glycerin is apparently related to the manure type and also time dependant due to the influence of the historical input on the result. International research indicates that there are differences regarding the optimal levels of glycerin additions. In general, it appears that adding a minimal amount up to 6% provides the best results.[17–20] A practical dilemma for the laboratory set-up was the twice per week feeding of the reactors which led to a relatively excessive addition of easily convertible VS of the glycerin into VFAs in the reactors. After the addition of the glycerin, the methanogens were not able to efficiently remove the sudden flux of available acetate and H_2_/CO_2,_ therefore, the methane content dropped directly after feeding but recovered within the same day and returned to higher levels before the next feeding (data not shown). This problem can be overcome in practice by feeding the glycerin more frequently in smaller doses which will prevent the drop in methane content. The rapid response of glycerin affords an opportunity to expoit the glycerin as a control mechanism for obtaining a certain level of biogas production. A relatively moderate amount of glycerin already results in reasonable biogas production.

Research into the codigestion of dairy cattle manure with 6% glycerin demonstrated that the best results were obtained in a retention time of 20 days at a loading rate of 2.9 kg VS/m^3^day which resulted in a specific methane yield of 0.60 m^3^ CH_4_/kg VS and a volumetric yield of 1.9 m^3^ CH_4_/m^3^_reactor_ per day for a CSTR, while the best results for an induced bed reactor (IBR) were obtained in a retention time of 18 days at a loading rate of 3.7 kg VS/m^3^day which resulted in a specific methane yield of 0.59 m^3^ CH_4_/kg VS and a volumetric yield of 2.0 m^3^ CH_4_/m^3^_reactor_ day.[21] In our research, less volumetric methane yield was attained but with a more moderate amount of added glycerine, and the retention time was shorter. These different results indicate that each case is unique and, therefore, each biogas plant is also distinct with its individualized specific configuration, manure quality/type and coproduct composition. To optimize a biogas plant for cost-effectiveness, the unique characteristics of the manure and coproducts as well as the changes over time in the compositions must be taken into consideration in order to be able to daily optimize the reactor for optimal performance and cost-effectiveness. The adaptive dynamic linear model employed in this experiment is able to perform such a job as was demonstrated in previous research.[8]

## 5. Conclusions

Decreasing the retention time of manure is possible; however, to which extent differed per manure type. For ‘low-quality’ swine manure, the retention time could be lowered to 15 days, but this resulted in lower biogas production per gram VS. For dairy cattle manure, the retention time could be decreased to 10–12 days, while the biogas production per gram VS even minimally increased. As expected, the addition of a moderate amount of easily biodegradable glycerin resulted in a substantial increase in biogas production. The biogas production almost triples at a retention time of 15.6 days with an addition of 4% glycerin. The relative production-enhancing effect of glycerin did not significantly vary for both manure types. However, the absolute production-enhancing effect of glycerin differed per manure type since the biogas production per gram VS also diverged per manure type. The statistical analysis of the performance of the reactors further indicated that the positive effect of the glycerin input diminished in shorter retention times. Therefore, the effect of glycerin addition depends on the manure type and retention time. In summary, by reducing the retention time and introducing glycerine, the volumetric biogas yield of the reactor of a manure-based biogas plant can be substantially increased. The ultimate effect of these measures depends both on the manure type and retention time.

## Appendix 1. Composition of the separate batches of manure and digestate.

**Table:**

			TS	VS	%VS	COD_t_	N_t_	VFA-COD	NH4-N	Density
	Days	pH (–)	(g/kg)	(g/kg)	of TS	(g/kg)	(g/kg)	(g/kg TS)	(g/kg)	(g/l)
Microferm digestate		8.06	54.0	37.6	69.6	52.1	5.33	6.7	3.85	1024
*Swine manure*										
Batch 1	0–31	7.21	67.8	52.9	78.0	73.1	4.09	90.8	2.66	1032
Batch 2	31–70	7.41	84.9	63.0	74.2	88.7	7.04	44.1	4.8	1046
Batch 3	70–98	7.83	78.0	54.1	69.4	81.9	6.19	24.6	4.0	1038
Batch 4	98–136	7.92	75.3	56.2	74.6	81.2	6.19	52.2	4.0	1015
Batch 5	136–144	7.32	82.8	57.8	69.8	82.6	7.39	10.6	5.0	1046.8
AVG		7.5	77.8	56.8	73.2	81.5	6.18	44.5	4.1	1035.6
STDEV.P		0.3	6.0	3.5	3.2	5.0	1.15	27.4	0.8	11.6
Dairy cattle manure digestate		7.82	53.2	40.0	75.2	61.7	3.49	16.0	1.98	1017
*Dairy cattle manure*										
Batch 1	0–31	7.46	77.8	61.2	78.7	96.9	3.56	105.5	1.74	994
Batch 2	31–66	7.46	84.2	65.7	78.1	103	3.58	181.3	1.6	999
Batch 3	66–98	7.03	89.4	71.3	79.7	101	3.58	123.3	1.7	1001
Batch 4	98–133	7.31	88.0	69.3	78.8	105	3.89	104.3	1.7	961
Batch 5	133–144	8.08	78.0	61.7	79.1	102	3.74	103.1	1.7	1014.3
AVG		7.5	83.5	65.9	78.9	101.6	3.67	123.5	1.7	993.8
STDEV.P		0.3	4.9	4.0	0.5	2.7	0.13	29.8	0.0	17.5
*Glycerin*		8.14	885.9	842.1	95.0	1185	0.76	40.5	<0.1	1351
